# Photoelectrode Durability in Two- versus Three-Electrode Configurations: Understanding the Impact of Circuit Configuration on Water-Splitting Stability

**DOI:** 10.1021/acsenergylett.6c00691

**Published:** 2026-04-23

**Authors:** Mitchell J. Hansen, James L. Young, Myles A. Steiner, Ryan O’Hayre, Todd G. Deutsch

**Affiliations:** † Advanced Energy Systems Graduate Program, 3557 Colorado School of Mines, Golden, Colorado 80401, United States; ‡ National Laboratory of the Rockies, Golden, Colorado 80401, United States; § Department of Metallurgical and Materials Engineering, 3557 Colorado School of Mines, Golden, Colorado 80401, United States

## Abstract

Device durability remains a significant challenge in photoelectrochemical (PEC) water splitting under ambient conditions. Yet, a lack of understanding of the test configuration and applied bias effects continue to hinder progress. In this study, we differentiate two-electrode (2E) and three-electrode (3E) configurations for evaluating PEC material durability, focusing particularly on their impacts on photoabsorber solid-state operating conditions. Our results underscore the fallacy of inferring 2E device stability from durability measurements performed solely in 3E configurations. Unmeasured and often misunderstood total circuit bias in 3E tests moderates material degradation, leading to the overestimation of photoelectrode stability compared to short-circuit operation. We demonstrate how the photoabsorber’s operating voltage critically governs charge separation, surface stability, and degradation mechanisms during PEC operation. With these findings, we propose a standardized framework for conducting more reliable 3E durability experiments that simulate unassisted performance to help accelerate the development of robust, stable materials for solar-driven water splitting.

## Graphical abstract

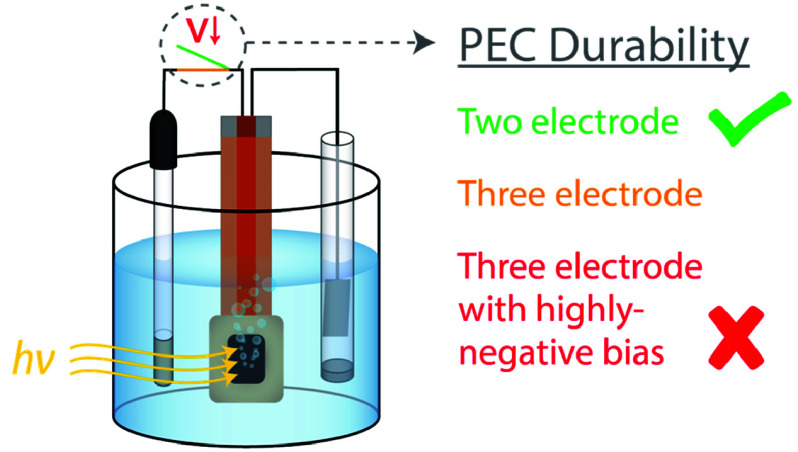

After the publication of seminal work by Fujishima and Honda in 1972, the field of photoelectrochemical (PEC) water splitting has generated an immense body of laboratory-scale research.^1,2^ However, even after over 50 years, there have been few demonstrations of this technology outside a laboratory setting,^3^ and the field still lacks rigorous standardization in testing and reporting for certain benchmarks. Extensive reports have been published on proper protocol for determining solar-to-hydrogen (STH) efficiency,^4,5^ that have helped facilitate vast improvements in overall device performance with landmark publications demonstrating nearly 20% STH efficiency.^6–8^ Fewer resources exist on proper procedure for PEC durability testing, yet device durability remains the biggest challenge for PEC water splitting, with the best devices lagging multiple orders of magnitude behind the goals necessary for application.^9^ The semiconductor materials used in high-efficiency photoelectrodes are prone to photocorrosion in aqueous electrolytes^10–12^ which has limited the most stable devices, operated in a relevant pH, to a maximum of 100 h of unassisted water splitting.^13–16^

A rigorous example of standardization in PEC water-splitting durability testing appears in the report published by Vanka et al.^17^ where the authors detail the recommended methods and equipment required to correctly measure photoelectrode stability. In that report, they highlight the importance of the PEC cell configuration in accurately determining cell durability. Landmark papers reporting devices with high STH efficiency have demonstrated the stark difference in two-electrode (2E) vs three-electrode (3E) measurements for assessing device durability.^6,7,18,19^ These reports demonstrate the propensity of 3E measurements to significantly overestimate the durability shown in the 2E configuration. However, the influence of test configuration on PEC durability is not common knowledge in the community, evidenced by a number of papers reporting stability metrics using only 3E measurements.^20–23^ Selected reports have proposed reasons such as greater charge separation and suppressed surface recombination for the improved lifetimes measured in 3E configurations,^6,19^ but there is no consensus on the exact causes of 3E experiments overestimating PEC durability.

The distinction between 2E and 3E experiments lies in the voltage reference of the circuit. It is common practice in electrochemistry to use a stable reference electrode (RE) with a known potential to enable accurate comparison of data across laboratories. This procedure works well for isolating the working electrode (WE) and characterizing its behavior with respect to its associated half-reaction, but it fundamentally cannot provide information on full-cell performance. Since the potential at the WE in 3E experiments is applied and recorded against a well-defined reference potential, and the counter electrode (CE) potential is not considered, performance of the CE does not affect results. Modern potentiostats are designed to apply the necessary potential to the CE, which is commonly not measured or reported, to provide an equal and opposite current of that at the WE. Therefore, 3E experiments are inherently assisted (a WE at 0 V vs RE has a circuit bias!) and do not provide representative information on the overall cell efficiency or durability. However, in 2E experiments, the bias applied at the WE is in reference to the polarized CE. Thus, the performance of the CE greatly affects the measurement,^17,24,25^ but the resulting data represent an accurate depiction of overall water-splitting metrics, where unassisted PEC experiments are performed at 0 V vs CE. Ultimately, we argue this difference in potential at the WE and its effects on the solid-state operating point of the photoelectrode dictates the stark differences in PEC lifetimes measured from 2E and 3E experiments.

In this Letter, we present a quantitative distinction between 2E and 3E PEC durability measurements from the perspective of the photoabsorber. We clarify how measurement configuration affects important stability metrics, and we use this information to offer guidance on the standardization of PEC durability experiments. We use a tandem GaInP/GaAs photocathode (Supplementary Figure S1) as our representative high-efficiency structure, and we employ a dual-working-electrode (DWE) architecture (Figure 1) to measure the photoabsorber operating characteristics. By measuring the surface potential at the top (T) contact while controlling the bulk potential at the rear (R) contact, we track the photovoltaic (PV) operating point during PEC operation for comparison in both 2E and 3E configurations. Overall, this work aims to establish the operating parameters required to properly assess PEC durability, even for materials that cannot split water unassisted.

**Figure 1:**
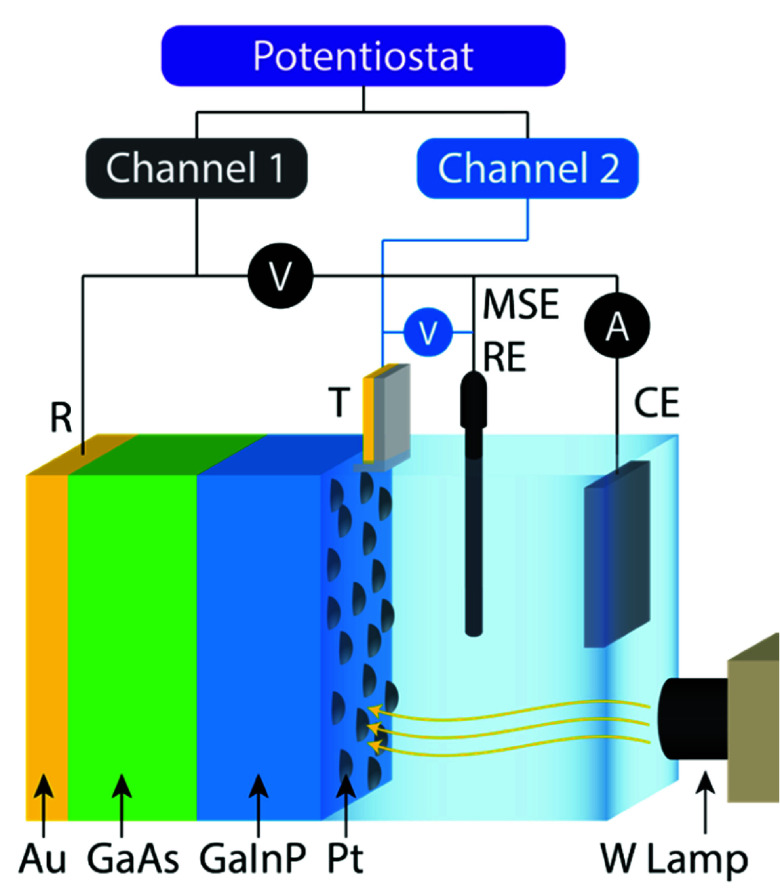
Schematic of DWE setup and potentiostat connections.

To represent a general photoabsorber capable of unassisted water splitting, we selected the buried-junction GaInP/GaAs tandem cell due to its high performance (STH efficiency ∼ 10%) and repeatable fabrication relative to other high-efficiency devices.^6,26^ The DWE structure required postgrowth processing including the electrodeposition of a gold back contact, electrodeposition of gold, front-contact busbars through a photoresist mask, followed by the deposition of Pt cocatalyst^6^ and finally the fabrication of photoelectrodes.^5^ The front contact of our DWE structure consists of only a gold busbar since this offers very similar potential sensing accuracy as the full metal grid contact commonly used in the PV community (Supplementary Figure S2).

We characterized the photoelectrodes using linear sweep voltammetry (LSV) and chronoamperometry (CA) in both 2E and 3E configurations in acidic electrolyte. The photoelectrode serves as the photocathode and the site of the hydrogen evolution reaction (HER) in all experiments, with the CE responsible for performing the oxygen evolution reaction (OER). To demonstrate the performance impacts of the CE, we use an IrOx on Ti mesh and a Pt flag which represent low-overpotential and high­(er)-overpotential OER catalysts, respectively. All photoelectrochemical experiments were performed using a two-channel Bio-Logic potentiostat with synchronized channels (Figure 1); the voltage-sense lead from channel two measures the open-circuit potential (OCP) of the photoabsorber top contact in reference to a Hg/HgSO_4_ (mercury/mercurous sulfate, MSE) RE.

Figure 2 demonstrates the effect of CE choice on photoelectrode performance in 2E and 3E configurations. The current density–voltage (*JV*) curves shown in Figure 2a reveal nearly identical performance despite the disparity in CE potential at any given point on the curve. This clearly demonstrates the absence of CE overpotential impacts on 3E measurements. However, the 2E measurements (Figure 2b) show a heavy influence from the CE overpotential. The difference in kinetic performance at the CE for Pt vs IrO*x* manifests as a shift in the photocurrent onset potential by 240 mV, and the resulting current measured at the short-circuit condition of 0 V vs CE differs by over 6 mA/cm^2^. Therefore, each measurement in the 2E configuration reflects the full-cell performance, but 3E measurements only characterize the half-cell performance of the WE and inherently do not accurately represent full-cell metrics, particularly STH efficiency. The 3E experiments mask the influence of CE performance on the full-cell current. Thus, all applied bias photon-to-current efficiency measurements using a RE in the 3E configuration represent only a half-cell efficiency and should be considered invalid if presented as an overall STH efficiency.^4,27^

**Figure 2:**
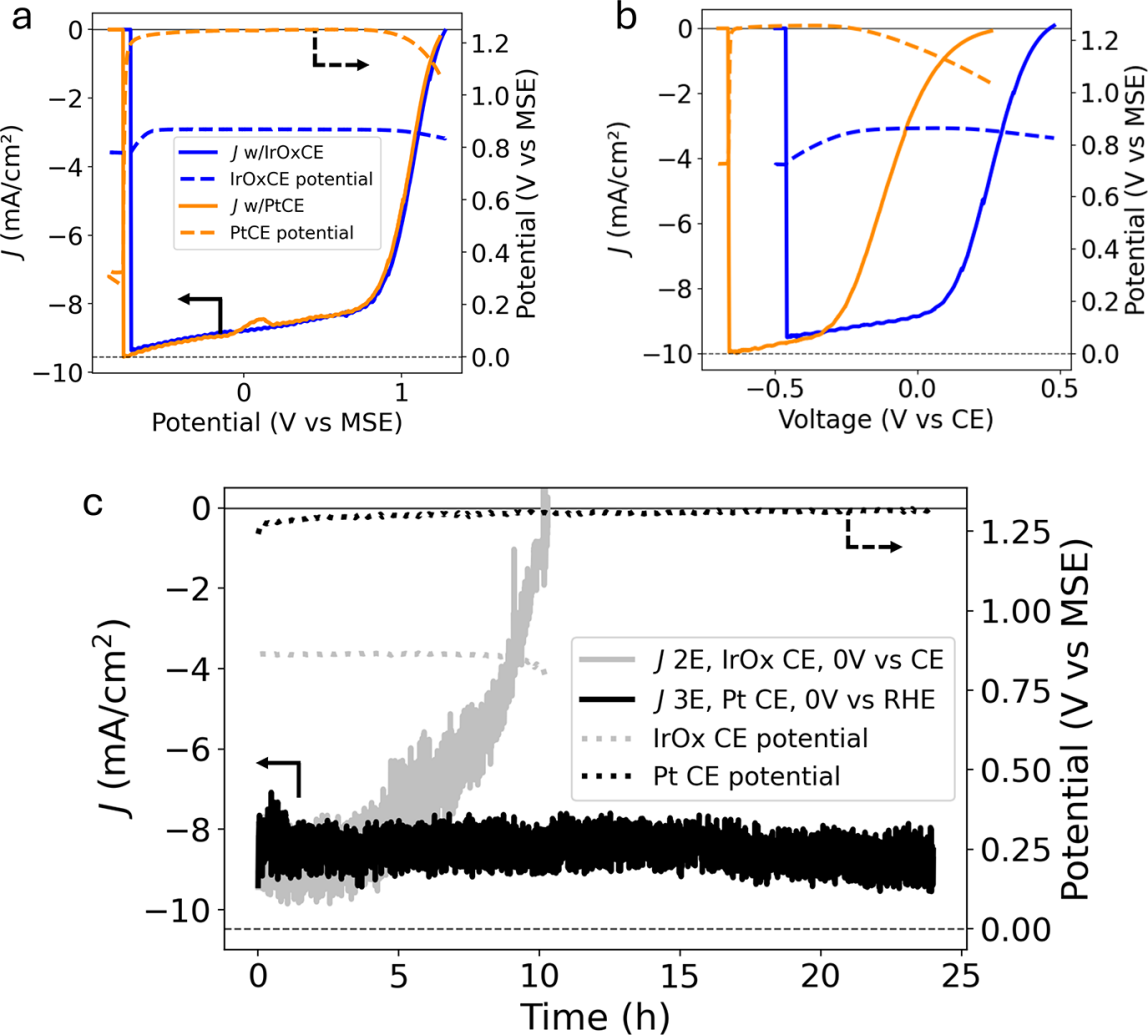
Comparison of (a) 3E and (b) 2E LSV data with different CEs, where *JV*s are measured from negative potentials to OCP; (c) Durability test (CA data) with overlaid CE potential for both 2E and 3E configurations. In all double-*y*-axis figures in this manuscript, solid lines represent the left *y*-axis and dashed lines go with the right *y*-axis.

The CA data in Figure 2c highlight the inability of 3E measurements to reliably characterize a material’s durability for unbiased PEC water splitting. Despite the commensurate starting current densities and lower CE and full-cell potential in the 2E configuration, the photoelectrode exhibits only 10 h of operation before reaching zero current, whereas the 3E experiments show steady current density for the full 24 h measurement. Therefore, other influences on photoelectrode durability must be evaluated. Here, we build on and investigate the hypothesis that improved charge separation provided by the externally applied bias in the 3E configuration increases durability.^6^ By monitoring the photoelectrode surface potential, we construct a quantitative picture of the photoabsorber bias during PEC operation.

In the buried-junction tandem-photoelectrode structure,^28^ the surface potential measured at the T contact represents the electrochemical potential of the photogenerated electrons (electron quasi-Fermi level), referred to as *V*_T_, providing insight into the PV performance during PEC testing. Figure 3a shows the *JV* curves from Figure 2a again, but the overlaid data (dotted lines) represents the surface potential (*V*_T_ vs MSE) rather than the CE potential. These data show that sweeping the R contact potential (*V*_R_) from negative potentials to OCP results in constant surface potential at the overpotential required to drive hydrogen evolution at the light-limited photocurrent of the photoelectrode until it increases near OCP. This behavior is also consistent with trends previously reported in literature.^29,30^ During durability testing, the surface potentials measured in the 2E and 3E configurations show similar values for the life of the photoelectrodes (Figure 3c, dotted lines). Despite this similarity in *V*_T_, the 2E measured photocurrent density quickly diminishes while the 3E measurement maintains a stable photocurrent density for 24 h. The macroscopic degradation phenomena are shown in time-lapse microscopy photos found in Supplementary Figure S4.

**Figure 3:**
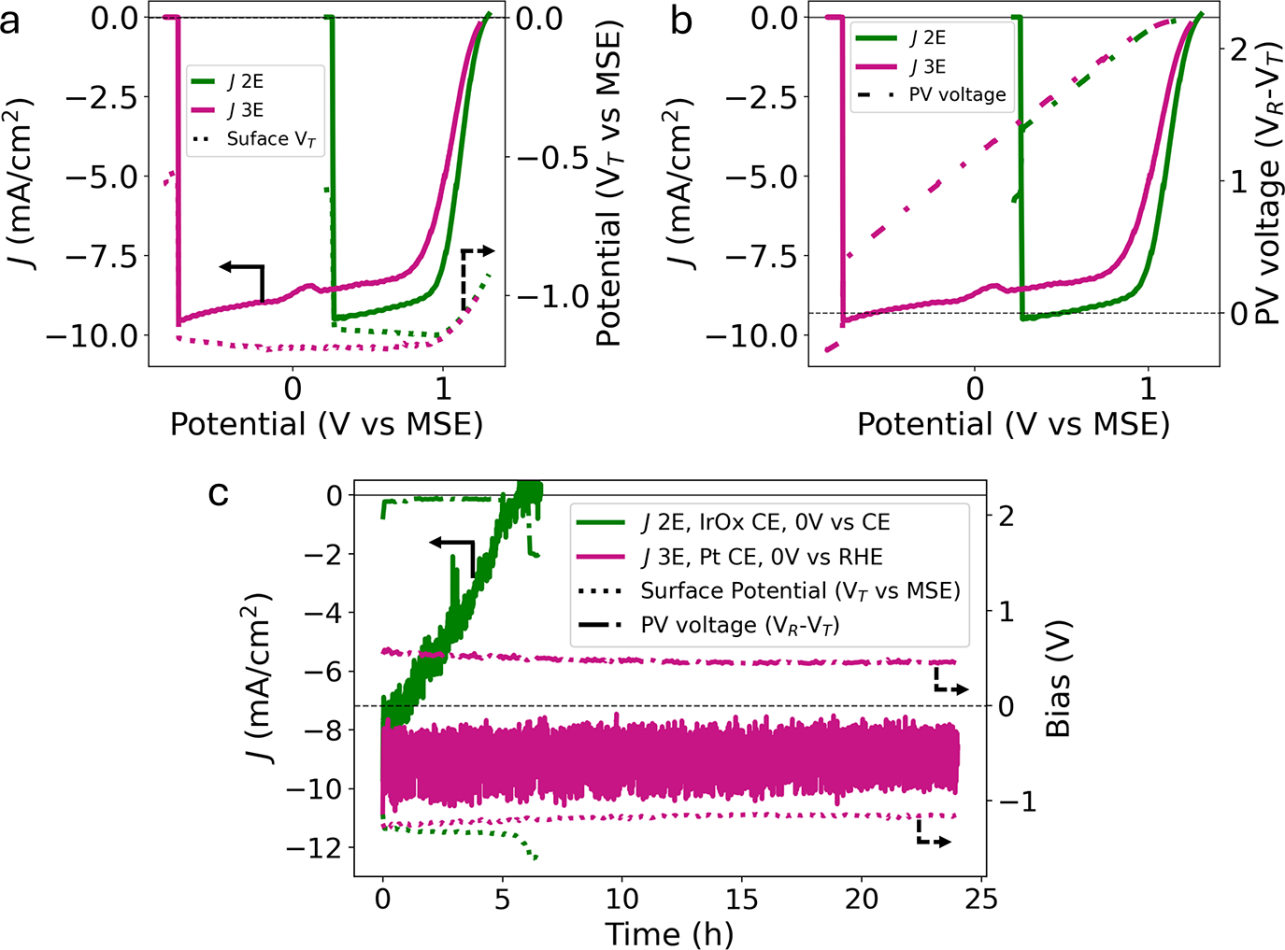
Comparison of surface potential and PV voltage for 3E and 2E measurements. (a) LSV data (solid lines) with overlaid surface potential (dotted lines) and (b) PV voltage (dashed lines). (c) Durability of 2E vs 3E with surface potential and PV voltage.

Using the surface-potential data, we can remove the reference electrode dependence by subtracting the front-contact potential (*V*_T_) from the back-contact (*V*_R_) potential to calculate the PV voltage. These solid-state data, shown in Figure 3b (dashed lines), demonstrate the increasing voltage provided by the photoelectrode while sweeping the R contact potential from −0.8 V vs MSE to OCP. The PV voltage provides more information than the raw surface potential data since photoelectrodes with nominally identical surfaces should produce the same current density at a given surface potential for a given reaction. The data shown in Figure 3a confirm that the T contacts measure very comparable potentials when operating anywhere in the light-limited photocurrent regime, regardless of CE material. Therefore, going forward, we use the PV voltage to characterize differences in PV operating conditions for 2E and 3E configurations.

The data in Figure 3c again show not only the decreased photoelectrode lifetime in the 2E versus 3E measurements, but they also reveal very different voltage conditions experienced by the PV. In the 2E measurements at 0 V vs CE, the photoelectrode experiences greater forward bias (operating near the PV open-circuit voltage) of over 2.1 V versus the roughly 0.5 V experienced in 3E measurements at 0 V vs RHE. The potentiostat provides the 1.6 V difference required to perform the reaction, helping to mask the photoelectrode degradation. This suggests that a higher PV photovoltage, and therefore greater forward bias, will accelerate photoelectrode degradation.

An analysis of tandem subcell operating points and band bending can illustrate how differing photovoltage and charge separation conditions between the 2E and 3E measurements influence durability. The total current and voltage of a series connected tandem can be understood in terms of each subcell’s operating parameters, where the total voltage is the sum of the subcell voltages, and the tandem current is limited to the lower of the two subcell currents. Importantly, the total PV voltage controls the band bending of the current-limiting junction (Figure 4a,b).^31^ By integrating the incident photon-to-current efficiency (IPCE) over the tungsten-halogen lamp spectrum, we show the devices used for this study are current limited by the top cell GaInP junction (Supplementary Figure S3). Therefore, during the 3E experiments at 0 V vs RHE, when the PV operates at a solid-state voltage near 0.5 V, the top cell experiences a mild reverse bias. This condition results in significant band bending at the top cell junction (Figure 4a), which creates a strong electric field that facilitates charge separation and cathodic protection by decreasing the flux of holes to the photoelectrode surface. In contrast, in the 2E measurements, the extra voltage required to drive the overall water splitting reaction results in the PV operating close to its open-circuit voltage, resulting in the top cell experiencing a significant forward bias. In this forward-bias condition, the top cell junction will exhibit decreased band bending (Figure 4b) which reduces charge separation and increases the flux of holes to the surface via diffusion.^32^ Since the III–V semiconductor materials are prone to oxidative corrosion in aqueous environments,^11^ this top cell forward bias may explain the lower lifetimes measured in the 2E configuration and confirm the importance of charge separation in controlling PEC durability.

**Figure 4:**
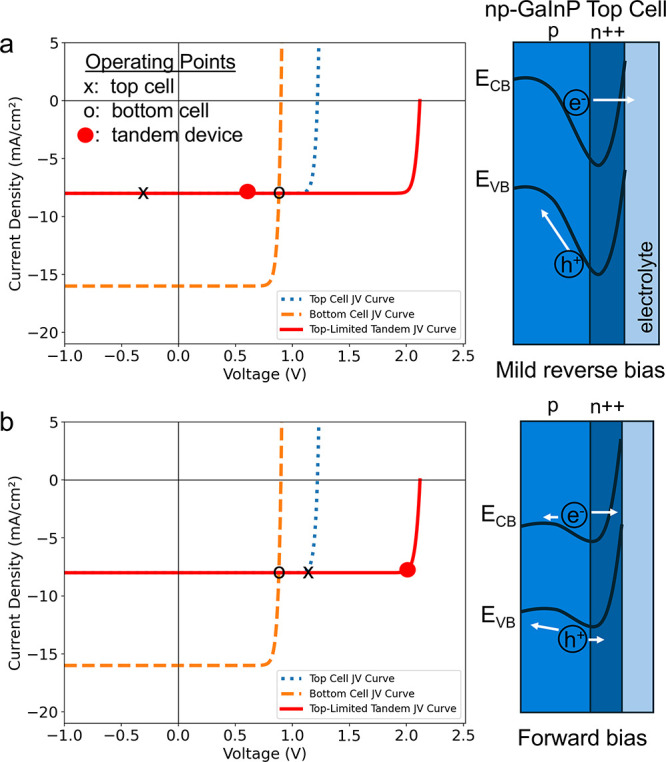
Tandem solid-state *JV* curves illustrating overall and subcell operating points with the corresponding qualitative top-cell band diagrams. (a) Conditions at 0.5 V PV voltage and (b) operating near solid-state open-circuit voltage.

To reinforce our conclusion that PV voltage significantly influences PEC durability, we conducted a 3E experiment at an applied potential where the PV experiences a similar bias to that of the 2E measurements. We used the DWE linear sweep voltammetry data to determine that 1.65 V vs RHE at *V*_R_ would result in the desired bias condition (Figure 5a). The results (Figure 5b) more closely mirror photoelectrode lifetime of the 2E configuration (Figures 2c and 3c). The current density decreases to 0 in 8 h, significantly shorter than the test at 0 V vs RHE, but within 1.5 h of the lifetime measured at 0 V vs IrO*x*. We repeated the durability tests in 2E and 3E configurations to obtain triplicates of each measurement, showing similar results (Supplementary Figure S5).

**Figure 5:**
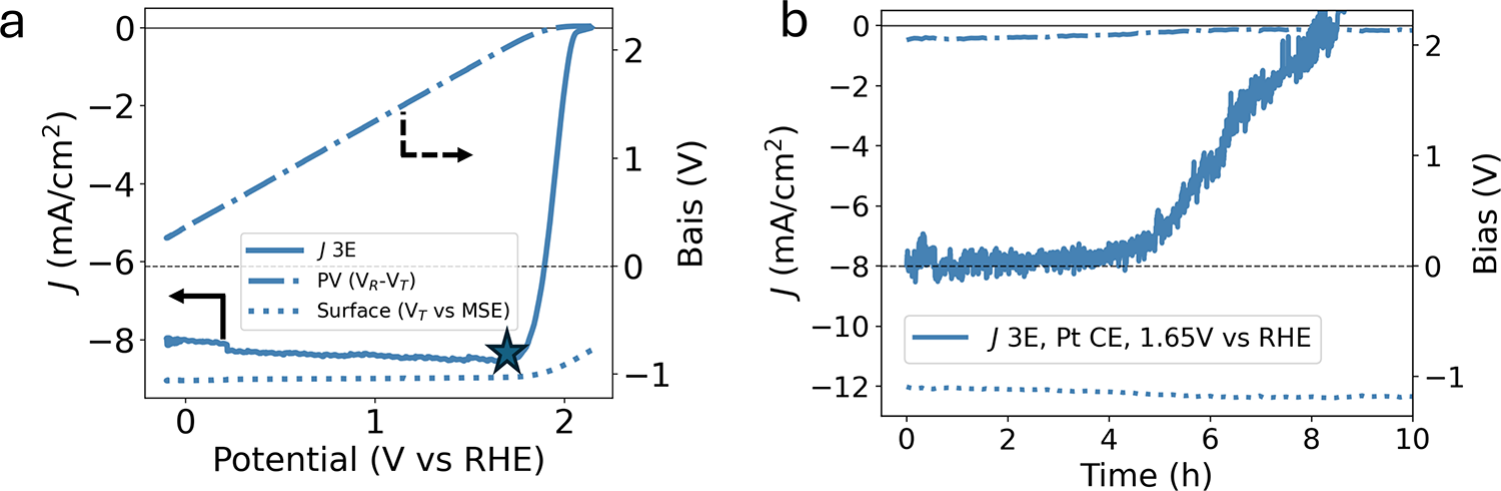
(a) *JV* curves for top-limited device in 3E configuration with overlaid PV voltage (dashed lines) and PEC surface potential (dotted lines). The star indicates the potential applied during the following (b) durability measurement.

To generalize these operating conditions for broad application, we must reintroduce the concept of the maximum power point (MPP) for PEC devices.^27^ While commonly used in the PV community, determining the MPP for a 3E PEC device can be elusive as “short-circuit” conditions are not well defined. Assuming close to ideal diode performance (i.e., free from current shunts that lead to a bias-dependent photocurrent in the light-limited regime), the MPP for 3E measurements is the maximum of −(*I* × (*V* – *V*_*SC*_)). *I* and *V* are the operating current and bulk potential, respectively, of the photoelectrode, and *V*_sc_ is the voltage at “short circuit,” which is the thermodynamic potential of the relevant water-splitting reaction (i.e., H^+^/H_2_ or O_2_/H_2_O for a photocathode or photoanode, respectively) (examples shown in Supplementary Figure S6). Importantly, the max power determined from 3E data should not be used to infer device efficiency.^27^ However, this calculation proves useful for determining a relevant operating regime for 3E durability testing.

By operating our photoelectrode at −0.1 V from its MPP, we enable more accurate predictions of unassisted photoelectrode lifetimes using 3E measurements.^6,33^ In contrast, applying 0 V vs RHE results in unrealistic reverse bias on the current-limiting top cell (and a total cell power output of zero), artificially enhancing charge separation and, thus, inflating photoelectrode durability. By replicating the PV voltage exhibited at short circuit in 2E measurements, we force the degree of charge separation to replicate the unassisted water-splitting conditions using the 3E configuration. It should be noted that the ideal applied potential for 3E durability measurements will depend on the specific overpotential conditions and kinetics associated with the intended WE and CE under study. In this work, the DWE architecture uniquely enables accurate determination of the bulk potential required to achieve the PV voltage that approximates 2E short-circuit conditions. However, if a given material makes the DWE architecture prohibitive, setting the bulk potential within 0.1 V of MPP will provide a more relevant operating point for durability testing than using 0 V vs RHE.

Although this study focuses on top-cell-current-limited tandem photoelectrodes, our recommendations for 3E durability measurement parameters still hold for bottom-cell-current-limited tandem photoelectrodes. The proposed relationship between top-cell forward bias and overall degradation rate would imply bottom-limited devices should demonstrate similar, diminished durability in either 2E or 3E configurations, regardless of applied bias. Future work will explicitly investigate the relationship between current-limiting junction and photoelectrode durability, but 3E measurements performed near the MPP will always provide more realistic durability information than tests conducted at some high applied bias. The recommendations of this work also apply to durability studies of half-cell photoanodes or photocathodes that are incapable of unbiased photoelectrolysis. Measurements conducted at a potential within 0.1 V of MPP will best characterize the durability of the photoelectrode under relevant bias conditions for materials integrated into an optimized unassisted device.^6^

Summary of measurement recommendations:

- Conduct unbiased durability measurements in 2E configuration, when possible
- Always perform 3E durability measurements within ±0.1 V of MPP
- Avoid drawing conclusions from 3E durability tests conducted at potentials far negative or positive of MPP for photocathodes and photoanodes, respectively

In conclusion, we use a DWE approach to provide the first quantitative and empirical support for the importance of test configuration on accurately measuring photoelectrode stability and efficiency. The 3E measurements only capture half-cell behavior, providing insight into photoelectrode performance but failing to predict durability or efficiency for overall water splitting. The external applied bias versus a stable reference in 3E measurements improves charge separation within the photoabsorber leading to cathodic protection. However, the presence of an applied bias in 3E measurements does not inherently improve these critical characteristics for stability. Rather, the magnitude of the applied bias and its effects on the PV operating voltage strongly influence stability. We propose that operating conditions which result in high forward bias on the topmost cell of a photocathode will allow hole transfer to the surface and promote oxidative corrosion, leading to decreased material durability. However, determination of the exact degradation mechanisms responsible for the shorter lifetimes will require further investigation. One may simulate full-cell operation by running 3E durability within 0.1 V of MPP. The experimental parameters required for the most accurate results will depend on the characteristics of the photoabsorber and the intended CE, but this operating range serves as a generalized standard to better capture true photoelectrode durability, even if a sample does not generate enough voltage to split water unassisted. We suggest these PEC durability measurement standards to permit more relevant comparison between different laboratories and facilitate accelerated development of more stable solar-water-splitting materials.

## Supplementary Material







## Notes

Sample fabrication, PEC characterizations, device structure and contacting scheme, IPCE, and photos and videos of electrodes during durability experiments (PDF)
Supporting video 1 - 2E durability timelapse (MP4)
Supporting video 2 - 3E durability timelapse (MP4)
