# Supplementary material for: Photoelectrode Durability in Two- versus Three-Electrode Configurations: Understanding the Impact of Circuit Configuration on Water-Splitting Stability
Source: ACS Energy Lett. 2026 Apr 23;11(5):4110–6. doi: 10.1021/acsenergylett.6c00691 (PMC13162313; doi:10.1021/acsenergylett.6c00691)
Supplement: Supplementary file 1 [file nz6c00691_si_001.pdf]

Photoelectrode Durability in Two- vs Three-Electrode Configurations: Understanding the Impact of Circuit Configuration on Water-Splitting Stability

Mitchell J. Hansen,† James L. Young,‡ Myles A. Steiner,‡ Ryan O'Hayre,¶ and Todd G. Deutsch*,‡

†*Advanced Energy Systems Graduate Program, Colorado School of Mines, Golden, CO 80401, USA*

‡*National Laboratory of the Rockies, Golden, CO 80401, USA*

¶*Department of Metallurgical and Materials Engineering, Colorado School of Mines, Golden, CO 80401, USA*

E-mail: Todd.Deutsch@nlr.gov

Experimental Methods

Sample Fabrication

Samples were grown by ambient pressure metalorganic vapor phase epitaxy on a custom-built reactor at the National Laboratory of the Rockies. The reagents included trimethylgallium, triethylgallium, trimethylindium, trimethylaluminum, arsine, and phosphine. Doping of the semiconductors was achieved using diethylzinc, disilane, dilute hydrogen selenide and carbon tetrachloride. The samples were grown on zinc-doped (001) GaAs substrates, miscut 2° toward the <111>B direction. All sources were mixed with a purified hydrogen carrier gas flowing at 6 LPM. The GaAs bottom cell was grown at 650°C,

the tunnel junction was grown at 600°C and the GaInP top cell was grown at 700°C. Growth rates ranging from 2 to 7 μm h^{-1}, and V/III ratios ranging from ~7 to ~200. After the growth of the top cell, the reactor was cooled to 650°C, and a GaAs front contact layer was grown to enable PV test structures and dual-working electrode (DWE) samples to be fabricated, and then the reactor was cooled to room temperature under an arsine overpressure.

After growth, the front epitaxial surface was protected by Shipley S1818 positive photoresist while the back of the substrate was cleaned in $NH_4OH:H_2O_2:H_2O$ (2:1:2 by volume, known as "212") before a gold back contact was electroplated onto each sample. To establish the front contact of the DWE structure, we used Shipley 1818 positive photoresist to form a mask and electroplated a 1 mm x 10 mm busbar consisting of a nickel adhesion layer and then ~1 μm gold. The remaining exposed GaAs contact layer was then removed using a 212 solution. Lastly, Pt co-catalyst was deposited via flash sputtering for 7 s while the busbars were protected using Kapton tape.

As stated in the main text, photoelectrode fabrication proceeded following the procedure outlined in previous work where the semiconductor was cleaved into roughly 5 mm x 5 mm pieces and mounted to copper tape on a glass microscope slide with silver paint (PELCO 16031).[1] An initial coat of epoxy was applied around the semiconductor sample and cured at 70°C for 30 minutes. Electrical contact to the front was established by painting silver from the busbars onto the epoxy and additional copper tape. Finally, Kapton tape and epoxy encapsulated the copper tape and the front contact to provide electrical insulation from the electrolyte. Electrode surface area was determined using an optical Keyence microscope (VHX-7000).

Photoelectrochemical Characterization

Photoelectrochemical (PEC) measurements used a Biologic SP-300 potentiostat and were conducted in a flat-faced glass cell filled with 0.5 M sulfuric acid (OmniTrace EMD

Millipore) electrolyte. Measurements were conducted in both two- and three-electrode configurations with a Hg/Hg_2SO_4 reference (Koslow Scientific) electrode (0.5 M H_2SO_4, +0.68 V vs SHE). IrOx on expanded Ti mesh (DeNora) and Pt flag counter electrodes were used, and the Pt counter electrode was contained in a glass tube with a frit to avoid contamination of the working electrode. Linear sweep voltammetry (LSV) was initially measured from negative potentials to OCV with a scan rate of 20 mV s^{-1}. All experiments used a 250-W tungsten-halogen light source (Newport Model #66883) equipped with a water-filled IR-absorbing filter and a light shaping diffuser (15° Newport 20DKIT-C2) in front of the sample, set to AM1.5G 1-sun intensity using a GaInP (1.81 eV bandgap) reference diode calibrated by NLR's Device Performance and Calibrations group. All PEC measurements were performed with the two channels of the bipotentiostat synced, with the second channel set to measure the OCP at the front contact of the photoelectrode. Macroscopic photoelectrode degradation was visually monitored during durability experiments using a digital microscope (Dino-Lite Edge Series AM7515MT2A).

Tandem Photoelectrode Architecture and Current Output

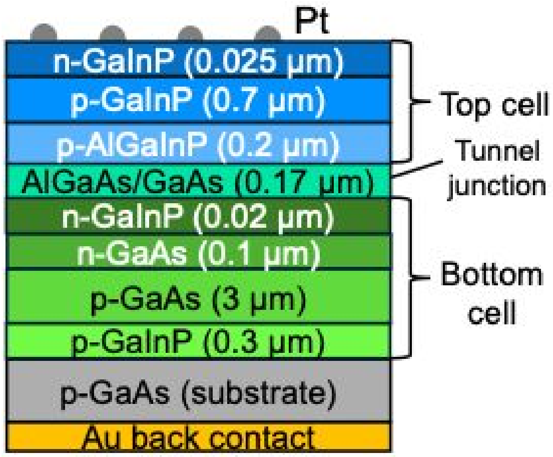

Figure S1: Schematic of semiconductor photoelectrode structure (run number MW707) after processing. Nominal layer thicknesses are indicated, not to scale.

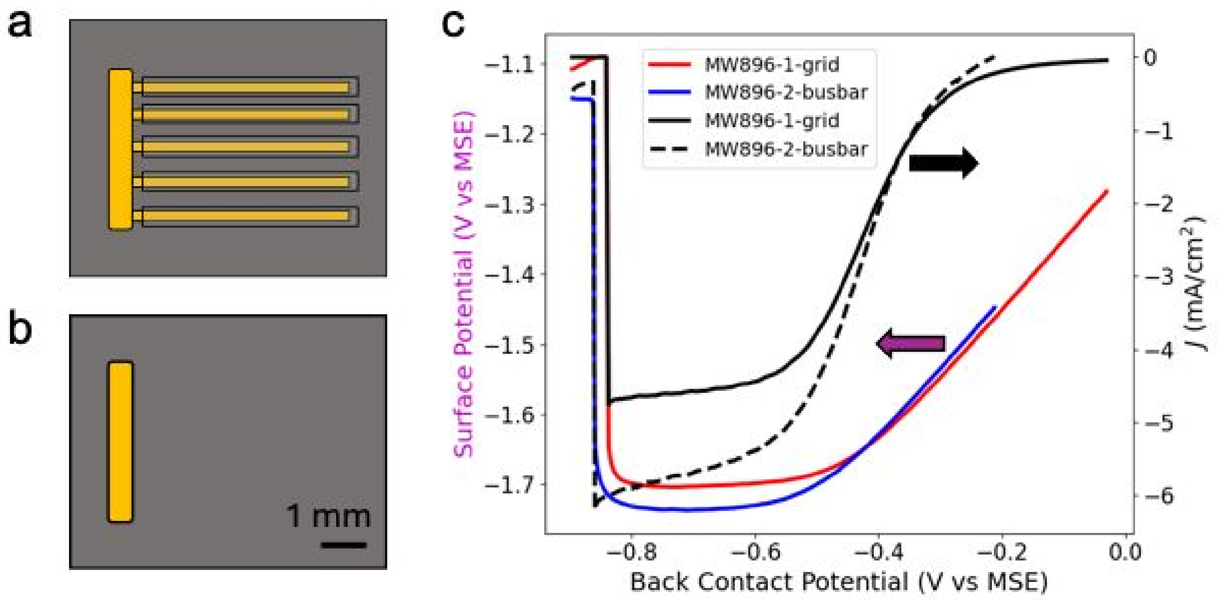

Figure S2: Comparison of potential sensing for two different DWE architectures. All exposed gold is eventually covered in epoxy during the electrode fabrication process. (a) Schematic of full grid front contact with a transparent SU-8 (Kayaku 2002) photoresist protecting gold fingers from electrolyte. (b) Schematic of busbar front contact (used in this work). (c) Results from LSVs of single, buried junction np-GaInP samples showing the front contact potential while sweeping the back contact potential. The close agreement between the potentials sensed by the full grid and only busbar (red and blue traces, respectively), at any given applied potential, demonstrate the efficacy of the busbar architecture to accurately measure the surface potential during PEC operation.

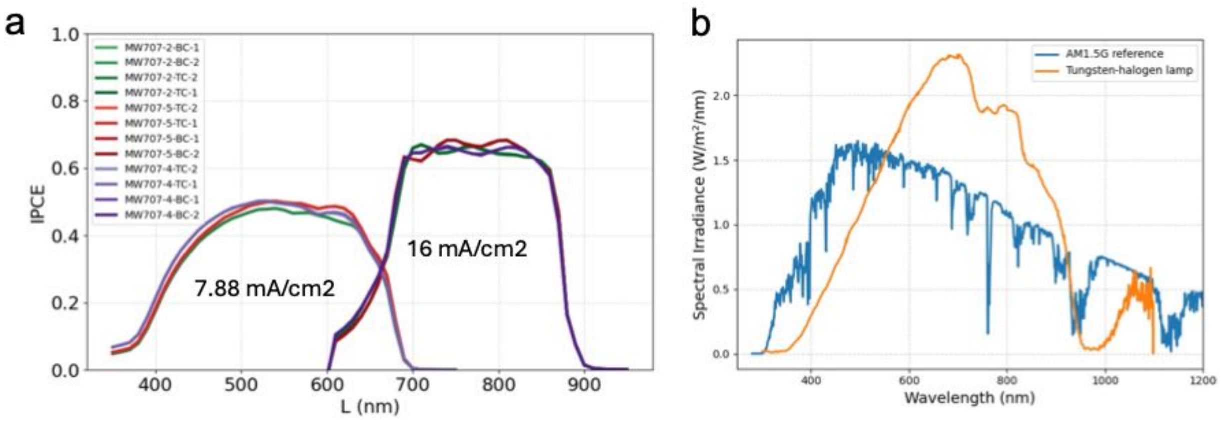

Figure S3: (a) IPCE data and integrated subcell current density results over (b) lamp spectrum

Supplemental Durability Data

a

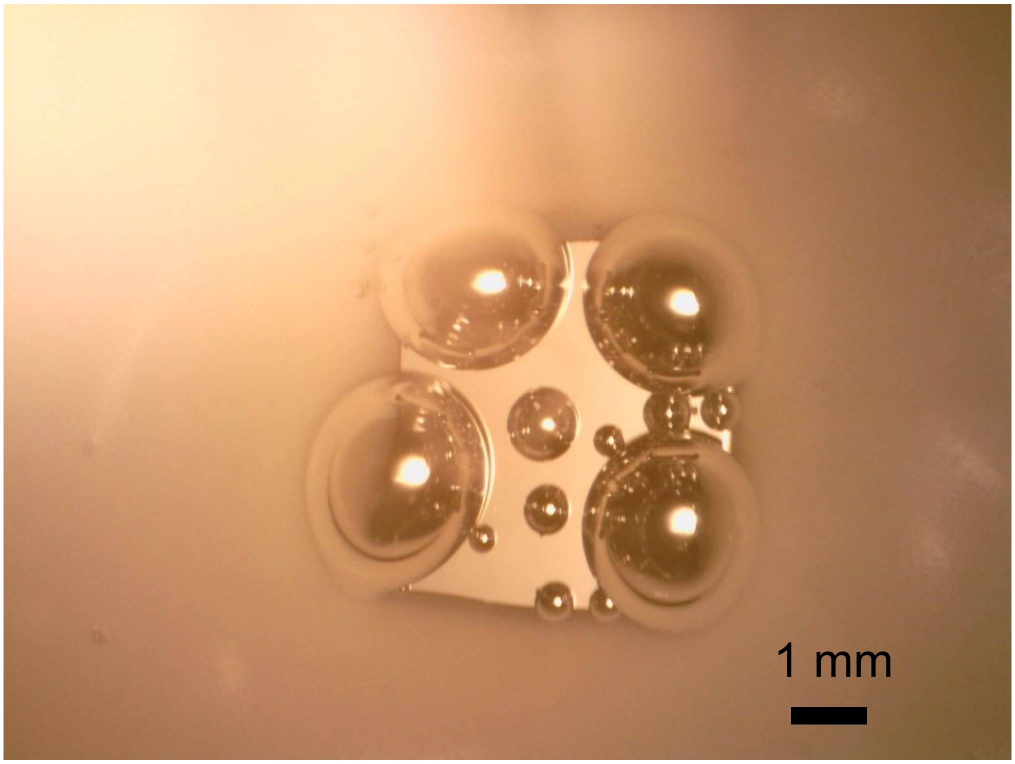

b

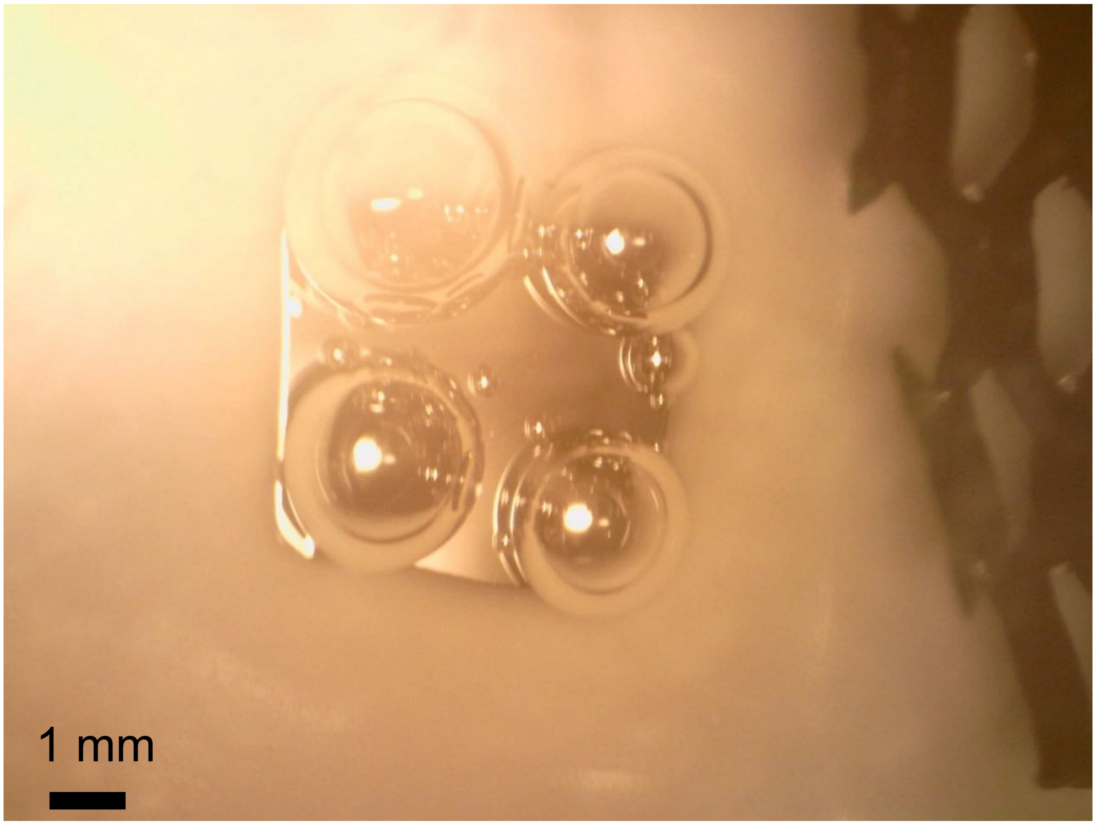

Figure S4: Screenshots from timelapse videos of photoelectrode degradation: (a) three-electrode, (b) two-electrode with part of the IrOx on expanded Ti mesh anode visible. Full videos included as supplementary files.

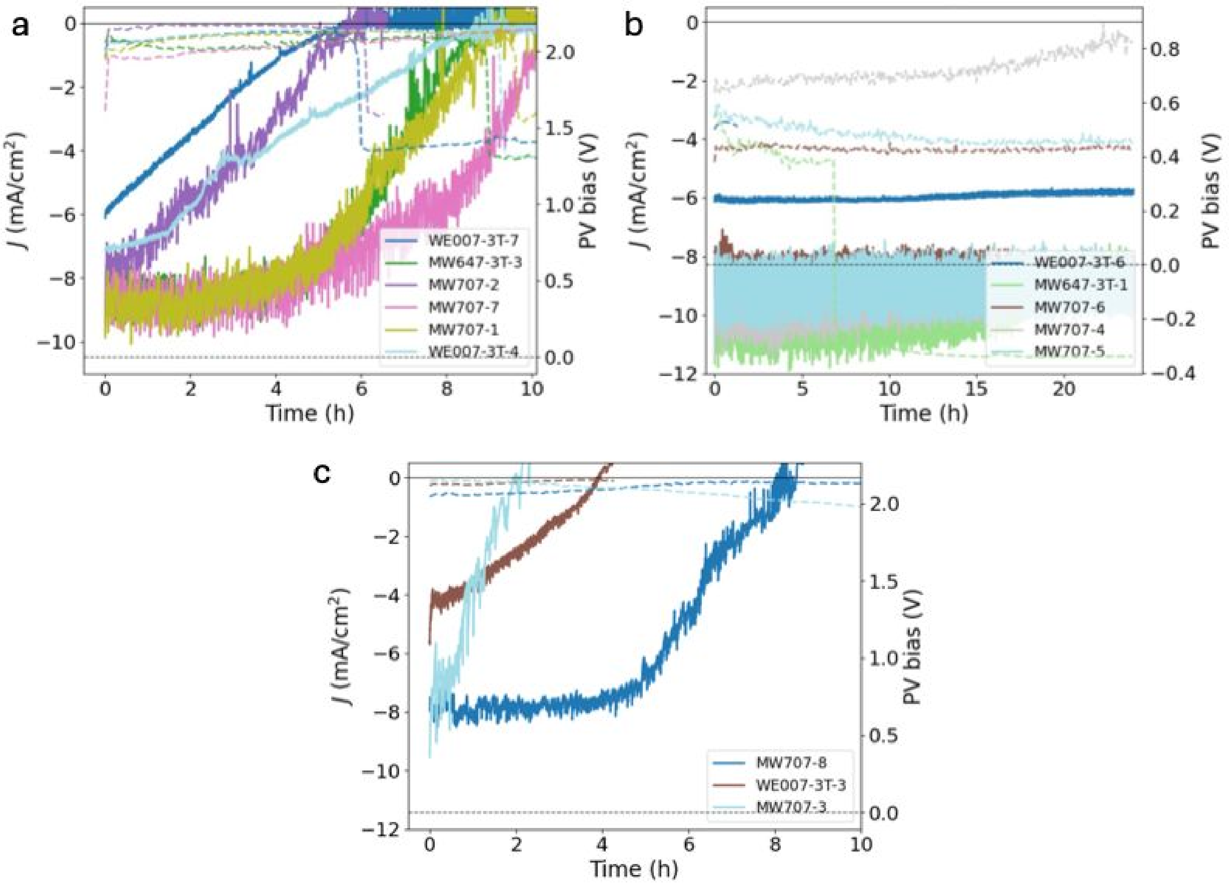

Figure S5: Replicates of durability measurements: (a) 2E at 0 V vs CE, (b) 3E at 0 V vs RHE, (c) 3E at 1.65 V vs RHE (-0.1 vs MPP).

Here, it is important to note that the variation in lifetimes within each category of experiments is likely due to variation in native and corrosion-induced defects.[2–4]

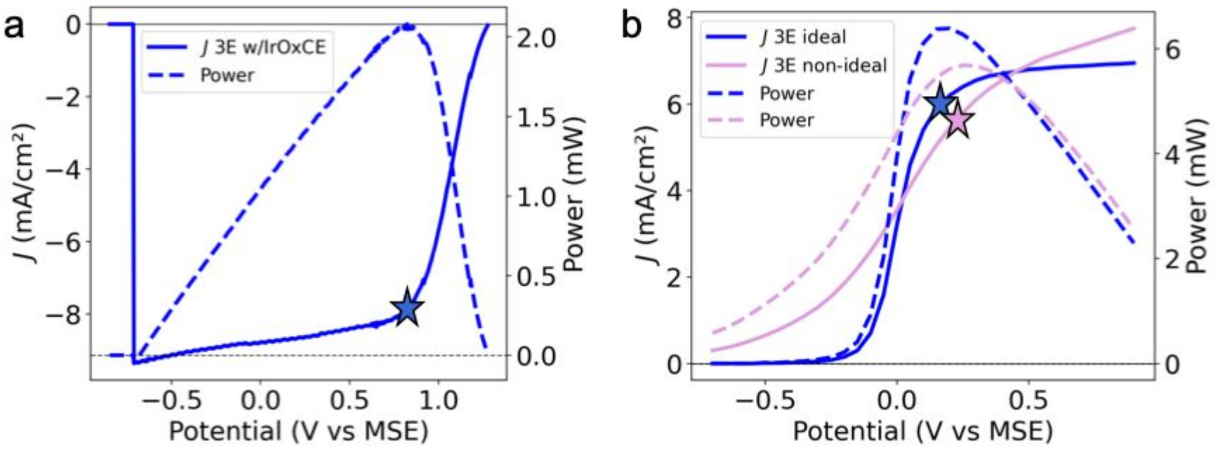

Figure S6: PEC power calculations using equations presented in main text for (a) 3E photocathode JV curve from Figure 2 and (b) example arbitrary 3E photoanode JV curves for near-ideal (blue) and non-ideal (light purple) diode performance. The stars in each panel indicate the MPP for the associated JV curve.

The width of the peak in the dashed purple trace in Figure S6 demonstrates how a device with high series resistance and low shunt resistance can have very similar power output at various operating points. This obscures the determination of MPP for the example device.

References

(1) Alley, O. J.; Wyatt, K.; Steiner, M. A.; Liu, G.; Kistler, T.; Zeng, G.; Larson, D. M.; Cooper, J. K.; Young, J. L.; Deutsch, T. G.; Toma, F. M. Best Practices in PEC Water Splitting: How to Reliably Measure Solar-to-Hydrogen Efficiency of Photoelectrodes. Frontiers in Energy Research 2022, 10, DOI: 10.3389/fenrg.2022.884364.

(2) Yu, W.; Buabthong, P.; Young, J. L.; Ifkovits, Z. P.; Byrne, S. T.; Steiner, M. A.; Deutsch, T. G.; Lewis, N. S. Failure Modes of Platinized pn+-GaInP Photocathodes for Solar-Driven H2 Evolution. ACS Applied Materials & Interfaces 2022, 14, Publisher: American Chemical Society, 26622–26630, DOI: 10.1021/acsami.2c01845.

(3) Moon, C.; Seger, B.; Vesborg, P. C. K.; Hansen, O.; Chorkendorff, I. Wireless Photoelectrochemical Water Splitting Using Triple-Junction Solar Cell Protected by TiO2. Cell Reports Physical Science 2020, 1, 100261, DOI: 10.1016/j.xcrp.2020.100261.

(4) Suresh Babu, D.; Schneider, S.; Rieth, T.; Sharp, I. D.; van de Krol, R. Unassisted PEC Water Splitting Using III–V Multijunction Photoabsorbers: Insights into the Degradation Mechanism. ACS Applied Energy Materials 2025, Publisher: American Chemical Society, DOI: 10.1021/acsaem.5c02884.
